# Equations for estimating binary mixture toxicity: Methyl-2-chloroacetoacetate-containing combinations

**DOI:** 10.1016/j.toxrep.2025.101939

**Published:** 2025-02-05

**Authors:** Douglas A. Dawson, T. Wayne Schultz

**Affiliations:** a Department of Biological Sciences and Toxicology, Ashland University, Ashland, OH 44805, USA; b College of Veterinary Medicine, University of Tennessee, Knoxville, TN 37996, USA

**Keywords:** Concentration addition, Independent action, Time-dependent toxicity, Electro(nucleophilic) reactivity, Mixture toxicity

## Abstract

Mixture toxicity was determined for 30 A+B combinations. Chemical A was the reactive soft electrophile methyl-2-chloroacetoacetate (M2CA), and chemical B was one of 30 reactive or non-reactive agents. Bioluminescence inhibition in *Allovibrio fischeri* was measured after 15-, 30-, and 45-minutes of exposure for A, B, and the mixture (MX) with EC_x_ (i.e., EC_25_, EC_50_, and EC_75_) values being calculated. Concentration-response curves (CRCs) were developed for A and B at each exposure duration and used to create predicted CRCs for the concentration addition (CA) and independent action (IA) mixture toxicity models. Likewise, MX CRCs were generated and compared with model predictions, along with the calculation of additivity quotient (AQ) and independence quotient (IQ) values. Mixture toxicity vs. the models showed various combined effects, including toxicity that was slightly greater than IA and/or CA, consistency with CA, IA or both models, effects that were less toxic than expected for either model and antagonism. Simple linear regression analyses of time-dependent toxicity (TDT) data showed very strong correlations (r^2^ ≥ 0.997) for B-TDT vs. the average TDT for A and B. Likewise, for both CA and IA, multiple linear regression analyses showed strong correlations (r^2^ > 0.960) between MX EC_x_ and either CA EC_x_ and AQ_x_ or IA EC_x_ and IQ_x_ values at each exposure duration. The results show that analyses of binary mixture toxicity data produced linear relationships resulting in equations that can effectively predict such toxicity.

## Highlights

• Single chemical and mixture toxicity was assessed at three exposure durations for 30 A+B M2CA-containing combinations.
• Combined effects varied from slight potentiation through additivity and independence to antagonism.
• Linear regression analyses of time-dependent toxicity (TDT) data showed very strong correlations between B-TDT and average A+B TDT values.
• Multiple linear regression analyses of toxicity data gave strong correlations for estimating binary mixture toxicity.
• The equations may be used for predicting binary mixture toxicity in Microtox for other such combinations.

## 1 Introduction

Research to improve the prediction and risk assessment of chemical mixture toxicity is ongoing. Recent advances have come in the areas of assessing chemical mixture toxicity and risks [1], risk evaluation and regulation [2], and risk assessment using dose-addition [3]. Other studies have developed a pathophysiology-based machine-learning system [4], a web-based tool box [5], or other computational methods [6].

Another approach being studied is using simple and multiple linear regression to develop equations for estimating binary mixture toxicity at multiple exposure durations [7]. In assessing mixture toxicity, concentration addition (CA) [8] and independent action (IA) [9] are frequently used to discern the combined effect of a given chemical mixture. While both models provide helpful context, binary chemical mixtures may produce a combined effect that is inconsistent with either model. For this reason, an approach that delivers high-quality equations for estimating binary mixture toxicity offers an opportunity to predict such toxicity better, thereby reducing the need for binary mixture testing [7]. The data derived from this approach may inform computational and read-across methods [10] used for predicting toxicity and enhancing modeling of the changing toxicity of binary mixtures over time.

Three terms are commonly used for interpreting the combined toxic effects of chemical mixtures: additivity, synergism and antagonism. Additivity has been used to note that the combined effect is as expected, whereas synergism indicates that the effect is more toxic than expected and antagonism indicates that the effect is less toxic than expected [11]. These combined effects can be further characterized. For example, additivity can be expressed as being additive by concentration/dose or additive by the magnitude of the response (i.e., effect summation). There are also different forms of synergism, such as co-synergy in which the chemicals enhance the effects of each other, and potentiation, in which one chemical increases the toxicity of another chemical without affecting the toxic endpoint itself [11].

However, one should note that it is not unusual for mixture toxicity to be additive at some point along a mixture CRC and either synergistic, antagonistic or both at other points. As a result, further characterization of the combined effect for a given mixture may be necessary. A case in point is for antagonism. For example, the combined effect of a binary mixture may actually be less toxic than expected by the CA or IA model, but can be either greater than or less than the toxicity caused by the more toxic of the two single agents. Hence, a more precise description of mixture toxicity associated with antagonism, as defined above, can be helpful.

Combined effects may also be described by the model of independent action [9]. It is based on probability theory for independent events and assumes that the components of the mixture do not interact with each other. With this model, the effect of two chemicals in combination should equal the sum of the effect of each chemical individually minus the product of their effects. In some cases, the combined effect of a mixture is well-modeled by IA.

Previous mixture toxicity studies using Microtox have evaluated combined effects from an electro(nucleo)philic reactivity perspective [12], [13], [14], [15], [16]. Electrophiles are electron-deficient chemicals that can undergo chemical reactions with electron-rich chemicals, i.e., nucleophiles. Within cells, exogenous electrophiles may react with endogenous nucleophiles, such as the N and O atoms in amino or nucleic acids, to form covalent bonds that lead to toxicity via, for example, enzyme inhibition or mutation. Reactivity mechanisms associated with organic chemicals include bimolecular (SN2) and aromatic (SNAr) nucleophilic substitution, Michael addition, and dehydration reactions leading to Schiff base formation.

The mixture toxicity studies with electrophiles noted above, included over forty individual chemicals. They were designed to examine mixture toxicity associated with electro(nucleo)philic reactivity. The intent was to mix or match reactivity mechanisms in binary combinations to discern whether a common reactivity mechanism would result in a consistent combined effect, such as CA, when compared with binary mixtures containing chemicals with different reactivity mechanisms. The latter might instead result in non-CA or, perhaps, IA effects. Binary combinations were selected because they would be the most likely to show a clear difference in mixture toxicity by reaction mechanism. Using more than two chemicals in mixtures would more likely mask any differences. In this report, the binary mixture results presented are limited to those that included M2CA as one of the two mixture components.

In this study, 30 binary combinations containing the soft electrophile methyl-2-chloroacetoacetate (M2CA) were tested to assess mixture toxicity and develop equations for estimating the toxicity of other such M2CA-containing combinations. This chemical is highly reactive with glutathione via SN2 substitution at the sp3 carbon atom [17], [18]. Selected for mixture testing due to that reactivity, M2CA tested alone in Microtox demonstrates a high degree of increasing toxicity over a 45-minute exposure period. The chemicals selected for testing with M2CA included those reactive by SN2, SNAr, Michael addition, and Schiff base formation mechanisms or for their lack of reactivity.

## 2 Materials and methods

### 2.1 Chemicals and reagents

Chemicals tested, with PubChem identification numbers, molecular weights, and known or putative chemical reactivity mechanisms [19], [20], [21] (Table 1), were purchased from Aldrich (Milwaukee, WI) or Sigma (St. Louis, MO) in high purity (≥95 %) and used without further purification. Dimethyl sulfoxide at ≤ 0.1 %, a non-toxic concentration, was used as the carrier solvent. Freeze-dried bacterial reagent, Microtox diluent, and the bacterial reconstitution solution were obtained from Modern Water (New Castle, DE). The bacterial reagent was kept frozen at −20°C before a 20-minute reconstitution period immediately prior to test initiation. Once that reconstitution period is completed, there is about a 1.5-hour period in which bacterial metabolism in the controls is stable. After that time metabolism begins to decline. For that reason, a new bacterial reagent vial, from the same lot, is needed for each test (i.e., A, B, and the mixture - MX) of the combination.

**Table 1:** Listing of chemicals tested.

Chemical	Abbr.	CID^a^	MW^b^	Mechanism of Reactivity^c^
2-butanone	2B	6569	72.11	Not reactive
Diethyl sulfate	DES	6163	154.18	Moderate SN2
3-methyl−2-butanone	3M2B	11251	86.13	Not reactive
Butyl glycidyl ether	BGE	17049	130.18	Epoxide SN2
Eugenol	EUG	3314	164.20	Not reactive^d^
Hydroxypropyl methacrylate	HPM	13539	130.14	Weak Micheal acceptor
Dimethyl sulfate	DMS	6497	126.13	Moderate SN2
3,4-hexanedione	34 H	62539	114.14	Dicarbonyl Schiff-base former
Methyl crotonate	MC	638132	100.12	Weak Micheal acceptor
β-propiolactone	BPPL	2365	72.06	Strained-ring acylating agent
Trichloroacetonitrile	TCLAN	11011	144.39	Haloform
Diethyl maleate	DEM	5271566	172.18	Moderate Micheal acceptor
Ethyl acrylate	EA	8821	100.12	Moderate Micheal acceptor
3-chloro−2-butanone^e^	3C2B	20026	106.55	SN2 – most probable mechanism
Methyl−2-bromopropionate	M2BP	95576	167.00	SN2; strong leaving, moderate activating
3-chloro−2,4-pentanedione^f^	3C24P	74328	134.56	SN2 – most probable mechanism
2,3-butanedione	23B	650	86.09	Dicarbonyl Schiff-base former
Ethyl chloroacetate	ECAC	7751	122.55	SN2; weak leaving, moderate activating
2-hydroxyethyl acrylate	2HEA	13165	116.12	Moderate Micheal acceptor
Ethyl vinyl ketone	EVK	15394	84.12	Strong Micheal acceptor
Methyl vinyl ketone	MVK	6570	70.09	Strong Micheal acceptor
4-nitrobenzyl bromide	4NBB	66011	216.03	SN2; strong leaving, strong activating
Bromoacetonitrile	BRAN	11534	119.95	SN2; strong leaving, weak activating
Methyl−2-chloroacetoacetate	M2CA	107332	150.56	SN2; weak leaving, strong activating
Ethyl propiolate	EP	12182	98.10	Strong Micheal acceptor
Chloroacetonitrile	CLAN	7856	75.49	SN2; weak leaving, weak activating
1-bromo−2,4-dinitrobenzene	BDNB	11441	247.00	SNAr; strong leaving and activating
Ethyl bromoacetate	EBAC	7748	167.00	SN2; strong leaving, moderate activating
1-chloro−2,4-dinitrobenzene	CDNB	6	202.55	SNAr, weak leaving, strong activating
Ethyl iodoacetate	EIAC	12183	214.00	SN2; strong leaving, moderate activating

a PubChem chemical identification number

b Molecular weight

c Chemical mechanism of reactivity was derived following the descriptions within [19], [20], [21]

d Requires metabolic activation for reactivity

e Has two reaction centers; hydrochlorination leading to a Michael acceptor is also a possible mechanism

f Has two reaction centers; both most likely result in SN2 products

### 2.2 Toxicity testing procedures

The marine bacterium *Allovibrio fischeri* was the model organism for testing. Toxicity, manifested as bioluminescence inhibition, was measured with a Microtox analyzer. The testing procedures for each binary combination included a test of each chemical individually (chemicals A and B) and the mixture (MX). Chemical A was always M2CA, while chemical B was one of the 30 chemicals noted in Table 1.

Each test within a given combination had seven duplicated concentrations and a duplicated control. Test concentrations, in mg/L, were prepared via serial dilution and later converted to µM. The dilution factor was the same for all tests of a given combination, but varied (1.75, 1.867, or 2.0) across combinations, primarily due to the change in toxicity over testing time for each B chemical. During testing, control and treatment vials were held at 15°C ± 0.2°C.

Initial light readings for each control and treatment replicate were taken before chemical addition. Toxicity assessments were made after 15-, 30-, and 45-minutes of chemical exposure. Microtox Omni software converted light readings to percent effect values.

### 2.3 Concentration-response curve (CRC) fitting and data calculations

Toxicity data were transferred to SigmaPlot (v. 15.0; Inpixon, Palo Alto, CA) for fitting to CRCs. User-developed program files fitted the raw data to sigmoid curves using the five-parameter logistic function from which the minimum effect parameter had been removed [14]. The four remaining parameters were maximum effect, EC_50_, slope, and asymmetry (s).

This modified function was designated the 5PL-1P (i.e., five-parameter logistic, minus one parameter) function to differentiate it from the software’s standard four- and five-parameter logistic functions. It was selected for use for several reasons. First, in previous mixture toxicity testing with two model organisms, the use of standard 3- and 4-parameter logistic functions, which lack an asymmetry parameter, was observed to result in single chemical and mixture CRCs that were often not symmetrical, thereby affecting precise determinations of EC_50_ and slope values. The five-parameter logistic function includes the asymmetry (s) parameter. However, to include it, the minimum effect parameter needed to be removed to reduce the likelihood of over-parameterization; that is, to minimize the number of situations in which not all parameters played a significant role in CRC development.

Additionally, these studies made use of an assay for which toxicity readings could be taken at multiple exposure durations, during which changing shapes of individual chemical and mixture CRCs were evident. Shape changes were especially noted for chemicals inducing toxicity that resulted, in whole or in part, from electro(nucleo)philic reactivity. Some chemicals showed reactive toxicity earlier in testing than others and that reactivity was more prominent at lower regions of the CRC for some chemicals than for others. Since both exposure duration and reactivity differences result in shape changes for single and mixture CRCs, use of the asymmetry parameter in CRC generation was supported.

With 5PL-1P being used for the analyses, curve-fitting used Eq. (1):

(1) $$y=\max/1+{\left(\frac{\mathrm{xb}}{x}\right)}^{\mathrm{Hillslop}e^{s}}$$

in which y = % effect, max = maximum effect, x = concentration, and s = asymmetry. The variable xb was determined using Eq. (2).

(2) $$\mathrm{xb}=\mathrm{EC}50\;\times 10^{\frac{1}{\mathrm{Hillslope}}}\times \log2^{\frac{1}{s}-1}$$

Initial parameters were estimated automatically. The constraints used for fitting were: a) EC_50_ > 0, b) 0.1 < s < 10, c) max = 100.

The following values were calculated for A, B and MX at each exposure duration: EC_25_, EC_50_, EC_75_, slope, s, maximum effect, and coefficient of determination (r^2^). For the B and MX tests, chemical concentrations were converted to M2CA-equivalents using the B factor (B_f_) from Eq. (3) [12]:

(3) $$\left[B_{f}\right]=\left[A\right]/\left[B\right]$$

Time-dependent toxicity (TDT) values were calculated by Eq. 4:

(4) TDT=((15minEC50−45minEC50)/(15minEC50×0.667))×100

to give a percentage-based value [7]. These calculations were made for A, B, and MX for each combination.

Calculation procedures for obtaining predicted CRCs for the CA and IA models have been described [13]. When A and B are equally effective in CA, the CA EC_50_ is left-shifted (when viewed graphically) by a dose-ratio (DR) factor of two. The CA_50_ and DR were calculated using (5), (6), respectively.

(5) CA50=a50/DR50

Herein, CA_50_ is the EC_50_ for CA, a_50_ is the EC_50_ of the more potent chemical, and b_50_ is the EC_50_ of the less potent chemical.

(6) $$\mathrm{DR}50=1+(\frac{a50}{b50})$$

Therefore, when a_50_ = b_50_ the DR_50_ = 1 + (1) = 2, that is, the CA_50_ = a50/2. This method allows one to calculate the predicted CA EC_50_ when A and B are not equally effective using the calculated DR to adjust the predicted CA_50_ value [7]. This approach was used to calculate EC_25_ and EC_75_ values for CA, thereby allowing the DR to be adjusted at various EC_x_ levels and, for example, in situations in which A was more potent than B at the EC_25_ and EC_50_, but less potent than B at the EC_75_. Taken together, the predicted CA values for each EC_x_, as well as the CA maximum effect value (Eq. 7) permit the calculation of the predicted CA curve using the 5PL-1P procedure noted above.

(7) max=a50×100x

Predicted curves for the IA model were developed using Eq. 8:

(8) $$\mathrm{yA}+(\mathrm{yB}\times \frac{100-\mathrm{yA}}{100})$$

with yA and yB being percent effect values for A and B, respectively.

For each combination and exposure duration, the three EC_x_ values were calculated for A, B, MX, and the predicted CA and IA curves. Concentration addition quotient (AQ) values were calculated via Eq. 9.

(9) AQx=MX ECx/CA ECx

Likewise, independent action quotient (IQ) values were calculated using Eq. 10:

(10) IQx=MX ECx/IA ECx

### 2.4 Assessment of mixture toxicity

Mixture toxicity assessments vs. the IA and CA models were made for each combination by initially determining whether the predicted 45-minute IA EC_50_ was either more toxic than, about equal to, or less toxic than that for CA. In this study, the mixture toxicity results presented are categorized by this metric, using the following steps and rationale, to characterize the combined effect of each combination more fully.

Herein, for a combination to be consistent with CA, IA or both, each of the three AQ_x_ or IQ_x_ values for that exposure duration must be within the range 1.00 ± 0.11 (i.e., 0.89 – 1.11). This range is used based on the collective results of more than 30 sham binary mixtures tested in these Microtox studies. Sham mixtures are those in which chemicals A and B are the same (e.g., M2CA:M2CA). A sham mixture should be CA, because by adding a given concentration of chemical B, one is actually just increasing the concentration of chemical A. In addition to calculating AQ and IQ values at the EC_50_, AQ and IQ values are calculated at the mixture EC_25_ and EC_75_. This is done to avoid concluding that a combined effect is CA or IA, simply because the mixture CRC happens to cross the predicted curve for the given model at the 50 % effect level, when it may deviate prominently from the model CRC elsewhere. So, visual comparisons of the entire mixture CRC relative to those for the CA and IA models are also used as they can be vital to adequately characterizing the combined effect.

Additionally, consideration of the mechanism(s) of reactivity for A and B is used to characterize combined effects. This is especially helpful when the predicted CRCs for CA and IA are close, resulting in AQ_x_ and IQ_x_ values that can both fall within their respective 1.00 ± 0.11 ranges. When this is the case and the two chemicals have the same reactivity mechanism, mixture toxicity is considered to be CA. In contrast, when the AQ_x_ and IQ_x_ values fall within the noted range and the two chemicals have different reactivity mechanisms, the combined effect is considered to be CA “coincident” and IA consistent.

When at least one of the AQ_x_ or IQ_x_ values falls outside 1.00 ± 0.11, mixture toxicity for a given combination is more fully characterized as follows. If one or more AQ_x_ or IQ_x_ value is below 0.89, the combined effect is considered to be “more toxic” than predicted by the model, whether it be CA, IA or both models. The term synergism is not applied unless the value is 0.75 or lower. The value point for this designation is arbitrary, but is used to indicate that toxicity was at a markedly lower concentration (i.e., more toxic) than expected for the CA or IA model. When one or more AQ_x_ or IQ_x_ value is above 1.11, the combined effect is at a higher concentration (i.e., less toxic) than predicted by the model.

Antagonism is applied to CA and IA for cases in which the mixture CRC, when viewed graphically, is at least partly located to the right of the CRC for the more toxic of the two single chemicals. This indicates the mixture is actually less toxic than the more toxic single chemical. There can also be instances in which the mixture CRC shows multiple combined effects. For example, the mixture CRC might show synergism along one stretch of the curve (e.g., below the EC_25_), cross over the CA and/or IA curve (e.g., between EC_25_ and EC_75_), and show antagonism along another stretch (e.g., above the EC_75_). In those situations, the combined effect of the combination is complex, so appropriate details are needed.

### 2.5 Data analyses

Herein, 30 binary combinations were tested; with M2CA serving as chemical A. One combination was a sham (M2CA-M2CA), giving 31 tests of M2CA alone. The repeatability of the M2CA tests was assessed by calculating the mean, standard deviation, coefficient of variation (CV), range, and Shapiro-Wilk W values for each EC_x_, slope, s, r^2^, and TDT value. The Shapiro-Wilk test evaluated the fitting of sample quartiles to standard normal quartiles [22].

Additionally, simple linear regression analyses were performed to discern any correlations between 45-minute MX EC_50_ values and 45-minute EC_50_ values for M2CA-alone, B-alone, and the AQ_50_ and IQ_50_ values. Linear regression was also used to assess correlations between TDT values for A, B, MX and the average of A+B (Avg. A+B TDT) for the combinations.

Equations were developed for use when estimating mixture toxicity for M2CA-containing binary mixtures not tested herein. Multiple linear regression (MLR) analyses utilized test data for MX EC_x_ as the dependent variable and either the CA EC_x_ and AQ_x_ or the IA EC_x_ and IQ_x_ as independent variables at each exposure duration. For each equation, r^2^, standard error of the estimate (SEE), and variance inflation factor (VIF) values were obtained. The VIF assessed collinearity between independent variables, with values < 5.0 having low concern for collinearity [23].

## 3 Results and discussion

### 3.1 Repeatability of M2CA data

The repeatability of the M2CA-alone tests was evaluated statistically (Table 2). All CV values for the 45-min EC_x_, slope, s, r^2^, and TDT values were below 31 and all but three were below 20, so the test-to-test variation was low [24]. In addition, the W statistic from the Shapiro-Wilk test denoted the fitting of sample quartiles to standard normal quartiles. Sample values with a W score = 1.0 represent a perfect fit [21]. For the M2CA-alone tests, W values were above 0.900 for each of the above listed measures, except for r^2^. Mean r^2^ values for these tests at each exposure duration were 0.9977 – 0.9991 (i.e., close to the upper limit of 1.0), leaving a narrow range for upper quartile values.

**Table 2:** Summary statistics for methyl-2-chloroacetoacetate (M2CA) tested alone (n = 31).

Parameter	Time (min)	Mean	Std. dev.	CV^a^	Range	SW-W^b^
EC_25_^c^	15	4.07	0.836	20.6	3.0	0.942
	30	1.84	0.334	18.1	1.2	0.956
	45	1.11	0.179	16.1	0.6	0.964
EC_50_^c^	15	9.64	1.885	19.5	6.6	0.937
	30	4.48	0.787	17.6	2.6	0.941
	45	2.74	0.439	16.0	1.6	0.959
EC_75_^c^	15	21.16	4.573	21.6	16.6	0.932
	30	9.59	1.803	18.8	6.0	0.933
	45	5.93	1.022	17.2	3.4	0.934
Slope	15	1.59	0.172	10.8	0.73	0.984
	30	1.75	0.147	8.4	0.61	0.981
	45	1.74	0.155	8.8	0.71	0.970
s^d^	15	0.69	0.210	30.3	1.03	0.906
	30	0.55	0.084	15.7	0.34	0.953
	45	0.52	0.079	15.1	0.30	0.946
r^2^ values	15	0.9977	0.002	0.17	0.009	0.659
	30	0.9988	0.001	0.07	0.004	0.830
	45	0.9991	0.001	0.06	0.003	0.875
TDT^e^	15–45	107.1	2.441	2.3	10.5	0.990

a CV: coefficient of variation = std. dev./mean * 100

b SW-W: Shapiro-Wilk test

c concentrations are M2CA-equivalent in µM

d s: asymmetry

e TDT: time-dependent toxicity value at EC_50_

### 3.2 Combined effects of M2CA-containing binary mixtures after 45-min

**Fig. 1:**
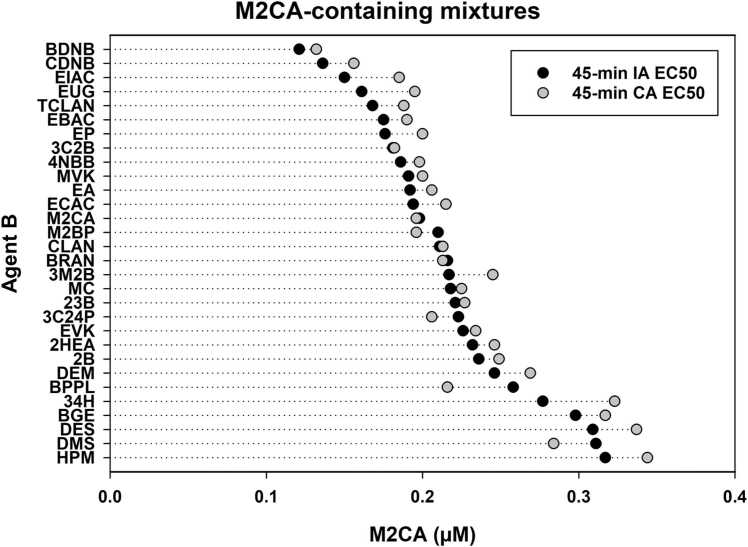
Comparative dot plot of predicted independent action (IA) and concentration addition (CA) EC_50_ values after 45-min exposures. Predicted IA toxicity was usually greater (i.e., at a lower M2CA-equivalent concentration) than predicted CA toxicity. The combinations are listed on the Y-axis simply as agent ‘B’ since M2CA was always agent ‘A’. The predicted IA EC_50_ values are plotted from most toxic to least toxic.

While combined effects were determined for each combination at each exposure duration, for brevity, only the 45-min results are presented. The combined effect of each combination after 45-minute exposures was initially assessed versus IA and CA. Combined effects determination for a given binary mixture can be more complicated when predicted IA EC_50_ and CA EC_50_ values are close to each other. To aid assessments, one can plot the predicted IA EC_50_ values, from most to least toxic combination, against the predicted CA EC_50_ values, to determine whether IA or CA represents the greater toxic hazard for each combination [7]. The dot plot (Fig. 1) shows that the predicted IA EC_50_ was more toxic than the predicted CA EC_50_ for 20 combinations. Six combinations had predicted IA EC_50_ and CA EC_50_ values that were within 0.05 µM of each other (i.e., these dots touch each other in the plot), while the predicted CA EC_50_ was more toxic for four combinations. Following that analysis, combined effects were assessed (as detailed above) by: a) whether or not 45-minute AQ_x_ and IQ_x_ values were 1.00 ± 0.11, b) via plots of MX CRC positioning relative to those for IA and CA, and c) whether or not A and B shared a common reactivity mechanism.

Two specific collections of cases are noteworthy from this approach. First, for the eight combinations in which each 45-minute AQ_x_ and IQ_x_ value was 1.00 ± 0.11, common or different reactivity mechanisms for A and B were used to designate the combined effects as being either CA or IA, respectively. Of the eight, six were designated CA, due to M2CA and B being SN2 reactive (Table 3). For the other two combinations, B was a Michael acceptor (M2CA-2HEA, M2CA-MVK), so these were designated IA (Table 3).

**Table 3:** 45-min M2CA-containing binary mixture toxicity designations vs. IA and CA models.

Mixture Toxicity vs. IA and CA^a^	Agent B^b^			
*IA EC*_*50*_*more toxic than CA EC*_*50*_*by at least 0.05 µM*^*c*^				
Less toxic than IA, slightly more toxic than CA	BDNB	CDNB		
Consistent with IA and CA	2HEA^d^	EBAC^e^	EIAC^e^	MVK^d^
Less toxic than IA; consistent with CA	4NBB^e^	TCLAN^e^		
Less toxic than IA; coincident^f^ with CA	EP^f^	EVK^f^		
Less toxic than IA and CA	3M2B EUG	DEM HPM	EA 34 H	ECAC DES
Antagonism (less toxic than M2CA alone)	2B	BGE		
*IA EC*_*50*_*≈ CA EC*_*50*_*(i.e., <0.05 µM apart)*				
Consistent with IA and CA	3C2B^e^	BRAN^e^	CLAN^e^	M2CA^e^
Less toxic than IA and CA	MC			
Antagonism (less toxic than M2CA alone)	23B			
*CA EC*_*50*_*more toxic than IA EC*_*50*_*by at least 0.05 µM*				
Slightly more toxic than CA and IA	3C24P			
Less toxic than CA; consistent with IA	BPPL			
Less toxic than CA and IA	M2BP	DMS		

^a^ IA – Independent action, CA – concentration addition

^b^ Mixtures are listed by chemical B abbreviation; chemical A was always M2CA

^c^ M2CA-equivalent concentrations

^d^ IA due to A and B having different reactivity mechanisms

^e^ CA due to A and B having a common reactivity mechanism

^f^ not CA due to agent B having a different reactivity mechanism than agent A

In the second set of cases, two combinations (M2CA-EP, M2CA-EVK) showed mixture toxicity that was slightly less toxic than predicted by IA but consistent with that predicted by CA. For these combinations, the combined effect was designated as “coincident” with CA because IA was the greater toxic hazard and B had a different reactivity mechanism than M2CA [7].

**Fig. 2:**
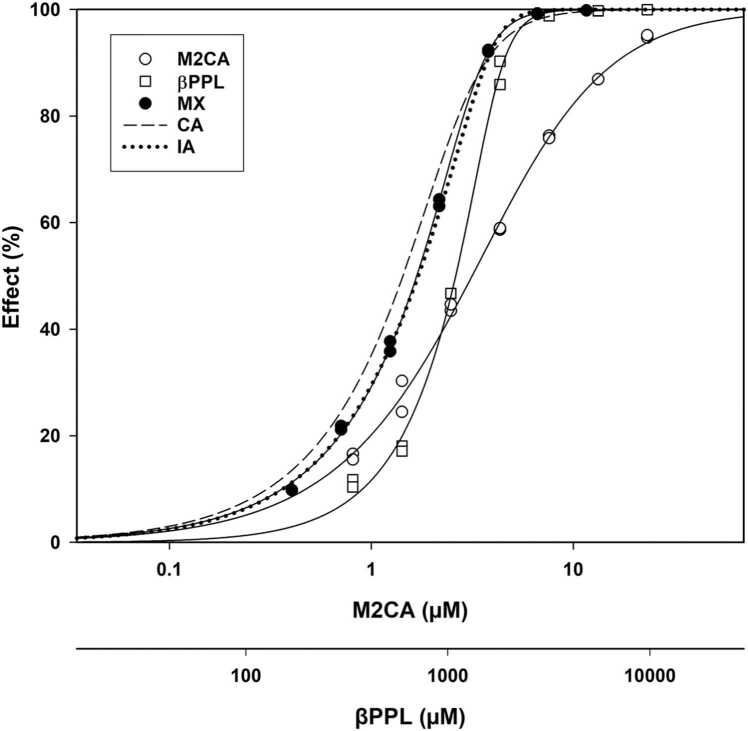
Concentration-response curve (CRC) plot for methyl-2-chloroacetaoacetate (M2CA) alone, β-propiolactone (BPPL) alone, the M2CA-BPPL mixture and the predicted concentration addition (CA) and independent action (IA) models. The BPPL data points are closely aligned with the predicted IA curve. All CRCs are given in M2CA concentrations (the upper X-axis). The lower X-axis depicts the CRC for actual BPPL concentrations.

**Fig. 3:**
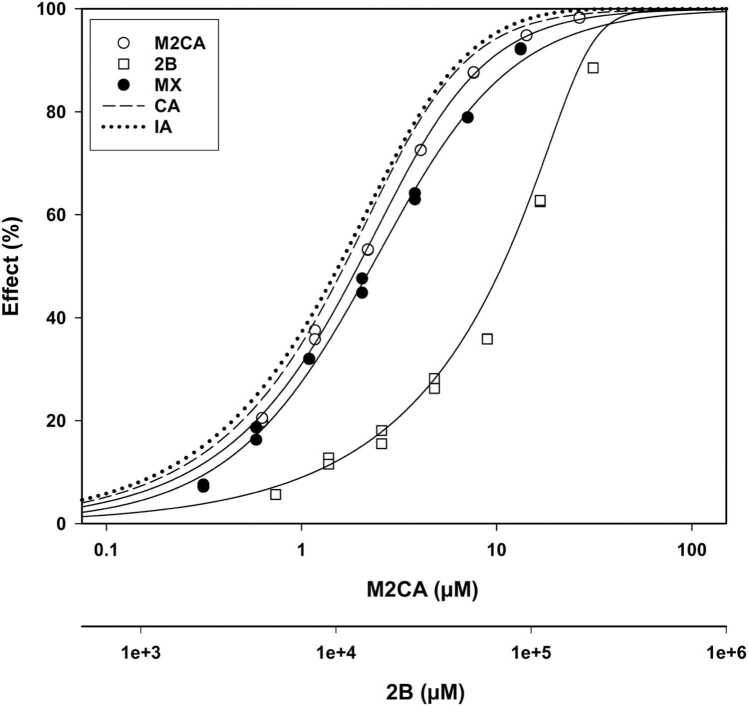
Concentration-response curve (CRC) plot for methyl-2-chloroacetaoacetate (M2CA) alone, 2-butanone (2B) alone, the M2CA-2B mixture and the predicted concentration addition (CA) and independent action (IA) models. The MX CRC is to the right of the M2CA-alone CRC, indicating the mixture was less toxic than M2CA, suggesting an instance of antagonism. All CRCs are given in M2CA concentrations (the upper X-axis). The lower X-axis depicts the CRC for actual 2B concentrations.

In the study, seventeen combinations contained a B that was either not reactive via SN2 or simply not reactive. Two were SNAr-reactive, eight were Michael acceptors, two were Schiff base formers, one was an acylating agent, one haloform reactive, and three were non-reactive. Based on this information, none would be expected to have had a combined effect consistent with CA although, as noted above, two were consistent with both IA and CA. Of the others, two were slightly more toxic than IA and CA, one was consistent with IA only (M2CA-BPPL – Fig. 2), while the rest had toxicity that was less toxic than predicted for IA, including three that were less toxic than M2CA alone – herein defined as antagonism – for which an example was M2CA-2B (Fig. 3).

**Fig. 4:**
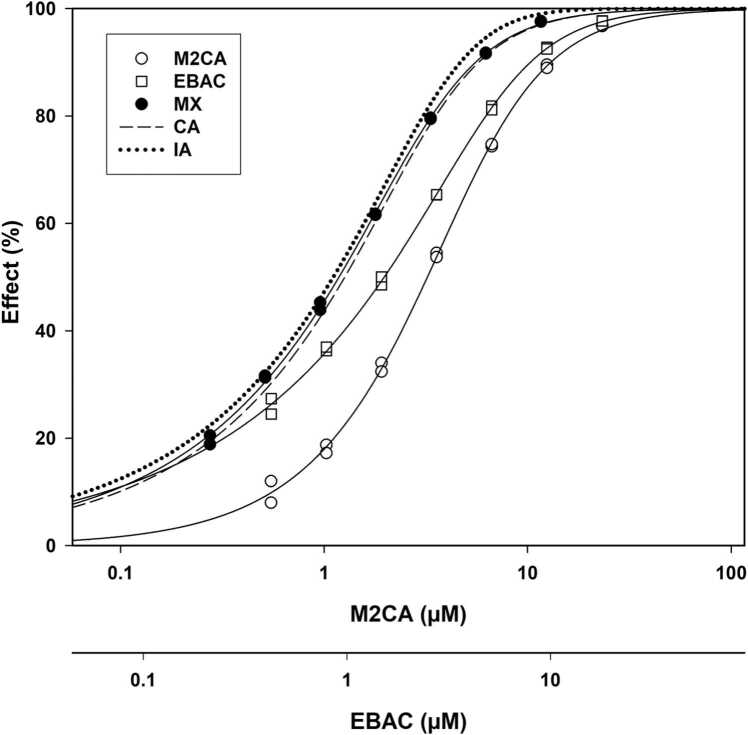
Concentration-response curve (CRC) plot for methyl-2-chloroacetaoacetate (M2CA) alone, ethyl bromoacetate (EBAC) alone, the M2CA-EBAC mixture and the predicted concentration addition (CA) and independent action (IA) models. The MX CRC is closely aligned with both the predicted IA and predicted CA CRCs. The combined effect was considered to be CA, since both M2CA and EBAC are reactive by the SN2 mechanism. All CRCs are given in M2CA concentrations (the upper X-axis). The lower X-axis depicts the CRC for actual EBAC concentrations.

For thirteen combinations, both chemicals had known or likely reactivity by the SN2 mechanism. Seven SN2 reactive B-chemicals scored as CA: 3C2B, 4NBB, BRAN, CLAN, EBAC (Fig. 4), EIAC, and M2CA (sham) after 45-minutes (Table 3). Three others had an AQ_50_ and one of the other two AQ_x_ values equal to 1.00 ± 0.11, while the third AQ_x_ was just outside that range: 3C24P (AQ_25_ = 0.86), ECAC (AQ_75_ = 1.18), and M2BP (AQ_75_ = 1.17). For practical purposes, these three combinations scored as approximately CA. Each of these combinations had B-TDT values > 60 %; thereby suggesting a substantial degree of electro(nucleo)philic reactivity for B during testing time.

The three SN2-reactive B toxicants that did not produce an approximately CA combined effect with M2CA, were the alkyl sulfates DES and DMS and the epoxide BGE. In these cases, all 45-minute AQ_x_ values were from 1.20 to 1.72 and the B-TDT values were below 10 %, suggesting weak reactivity at best. Weak reactivity may explain why these three combinations had a combined effect that was less toxic than predicted by CA, as follows. All chemicals, even highly reactive ones, exert baseline toxicity (i.e., narcosis) at sufficiently low concentrations. For SN2-reactive chemicals with weak reactivity, the toxicity observed would be predominantly narcosis-derived early in testing, especially at low concentrations. As exposure duration increased, reactivity-derived toxicity would become somewhat more prominently intermingled with baseline toxicity, especially at higher concentrations. In contrast, M2CA (mean TDT – 107 %) was more likely to have predominantly reactivity-derived toxicity at most or all tested concentrations over most of the exposure period.

### 3.3 Mixture EC_50_ comparisons with the EC_50_ for A, B, and the AQ and IQ values

Toxicity was determined for three exposure durations (15-, 30-, and 45-minute). However, detailed results are presented for just the latter timepoint. For each combination, 45-minute EC_50_ values for MX, A, and B (the latter given as both M2CA-equivalent and actual B concentrations), and the calculated 45-minute AQ_50_ and IQ_50_ values are presented (Table 4). Simple linear regressions conducted for the MX EC_50_ values vs. the respective values from each of the other data columns resulted in r^2^ values < 0.800, indicating some weak correlations but none of high enough quality for predictive purposes (i.e., ≥ 0.900).

**Table 4:** 45-min toxicity and quotient values for each combination.

Agent B	MX EC_50_^a^	A EC_50_	AQ_50_^b^	B EC_50_	B EC_50_^c^	IQ_50_^b^
2B	2.30	1.96	1.39	10.69	83,659.1	1.47
DES	2.98	3.15	1.33	7.77	325.2	1.46
3M2B	1.91	3.52	1.17	3.02	451.8	1.32
BGE	3.47	3.23	1.65	6.03	3489.6	1.75
EUG	1.44	2.77	1.11	2.44	19.2	1.34
HPM	2.62	3.31	1.15	7.39	3666.3	1.25
DMS	2.58	2.78	1.37	5.88	301.0	1.25
34 H	3.09	3.01	1.44	7.50	1977.6	1.68
MC	2.01	2.26	1.35	4.40	1984.6	1.39
BPPL	1.68	3.16	1.17	2.62	1093.7	0.98
TCLAN	1.30	2.59	1.04	2.42	5.7	1.17
DEM	2.08	3.34	1.16	3.83	239.3	1.27
EA	1.62	2.15	1.18	3.77	993.2	1.27
3C2B	1.21	2.76	1.00	2.14	108.1	1.00
M2BP	1.40	2.60	1.07	2.61	101.0	1.00
3C24P	1.26	2.86	0.92	2.62	8.4	0.85
23B	2.77	2.15	1.84	5.04	3526.4	1.89
ECAC	1.50	2.97	1.05	2.75	290.0	1.16
2HEA	1.58	3.40	0.97	3.14	582.1	1.02
EVK	1.56	2.70	1.00	3.66	2.9	1.04
MVK	1.32	2.26	0.99	3.21	2.76	1.04
4NBB	1.44	2.44	1.10	2.85	1.0	1.17
BRAN	1.43	2.43	1.01	3.39	7.4	1.00
M2CA	1.29	2.52	0.99	2.70	2.7	0.98
EP	1.24	2.36	0.93	3.03	5.8	1.06
CLAN	1.32	2.40	0.93	3.44	600.0	0.94
BDNB	0.84	2.47	0.95	1.36	2.3	1.04
EBAC	1.22	3.22	0.97	2.07	1.6	1.05
CDNB	0.91	2.13	0.88	2.02	6.01	1.00
EIAC	1.10	3.33	0.89	1.94	0.2	1.10
r^2^ vs. MX EC_50_^d^	----	0.067	0.776	0.662	0.563	0.707

^a^ concentrations are given as M2CA-equivalents in µM – except as noted

^b^ values are unitless

^c^ actual B-alone concentrations (µM) before conversion to M2CA equivalents

^d^ coefficient of determination vs. MX EC_50_

### 3.4 Analyses of time-dependent toxicity (TDT) data

Toxicity data at each exposure duration were collected and used to generate TDT values for A, B and MX for each combination (Table 5; note in Table 1, Table 4, Table 5 that the chemical listings are arranged in the same order, matching the order of TDT values for B from least to greatest). The TDT data for A (A-TDT), B (B-TDT), the average TDT for A and B of each combination (Avg. A+B TDT), and the mixture (MX-TDT) were analyzed by linear regression. To discern trends the data were analyzed collectively and in smaller sets based on whether or not the B chemicals were reactive and, if so, by their reactivity mechanism(s).

**Table 5:** Time-dependent toxicity (TDT) values per combination.

Agent B	A-TDT^a^	B-TDT^b^	Avg. A+B TDT^c^	MX-TDT^d^
2B	106.8	−28.8	39.0	69.5
DES	109.0	−18.2	45.4	62.2
3M2B	110.6	−13.3	48.7	34.5
BGE	110.5	−0.1	55.2	65.5
EUG	108.8	0.4	54.6	56.3
HPM	106.5	1.5	54.0	49.3
DMS	105.8	9.2	57.5	60.5
34 H	104.5	9.4	57.0	67.1
MC	105.1	18.6	61.9	67.4
BPPL	112.0	21.4	66.7	43.3
TCLAN	102.7	29.1	65.9	63.5
DEM	110.5	39.3	74.9	76.9
EA	106.1	54.1	80.1	80.8
3C2B	108.2	65.7	87.0	82.2
M2BP	104.9	70.2	87.6	87.7
3C24P	106.9	84.2	95.6	96.7
23B	106.7	85.1	95.9	91.5
ECAC	109.7	89.8	99.8	101.3
2HEA	105.3	94.3	99.8	104.0
EVK	107.7	95.1	101.4	100.0
MVK	107.8	97.1	102.5	100.9
4NBB	106.6	98.9	102.8	105.8
BRAN	104.6	101.2	102.9	104.7
M2CA	105.6	101.5	103.6	104.0
EP	108.2	105.5	106.9	109.3
CLAN	103.9	106.7	105.3	105.0
BDNB	109.4	113.2	111.3	105.0
EBAC	108.6	113.4	111.0	112.8
CDNB	106.3	121.1	113.7	110.2
EIAC	108.8	122.5	115.7	118.8

a TDT for chemical A (always M2CA)

b TDT for chemical B in order from least to greatest TDT

c Average A+B TDT = (A-TDT + B-TDT)/2

d TDT for the mixture

In all sets, A-TDT was not correlated with any other TDT grouping, as r^2^ values were always < 0.310 and < 0.010 for 25/28 (89 %) analyses. This is reasonable because M2CA-TDT values for all combinations had a small range of 10.5, while TDT value ranges for the other groupings were larger; B-TDT = 151.3, Avg. A+B TDT = 76.7; and MX-TDT = 84.3.

**Fig. 5:**
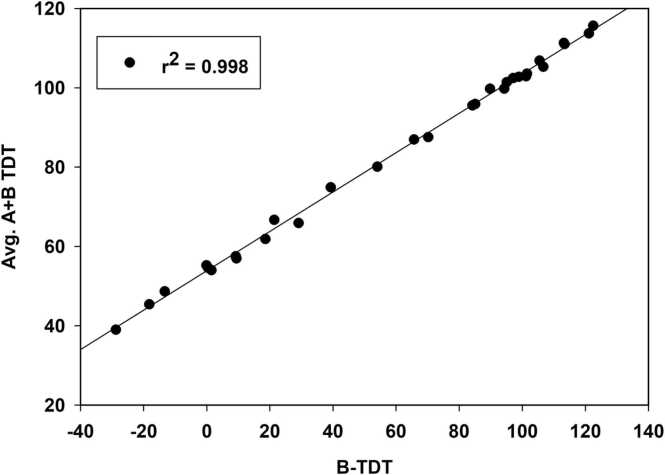
Linear regression plot of the time-dependent toxicity value for each chemical B vs. the average TDT value for A + B for each combination.

The Avg. A+B TDT data were very highly correlated with B-TDT data for the full data set and all subsets examined. The predictive equation for all combinations was: Avg. A+B TDT = 53.843 + (0.497 * B-TDT); n = 30, r^2^ = 0.998, SEE = 1.131, p < 0.001 (Fig. 5). In a previous study of 150 binary combinations that mixed and matched various electrophiles and some non-reactive chemicals, it was shown that one could estimate the MX-TDT for a given mixture by averaging the A-TDT and B-TDT values [25]. So, conceptually, when the A-TDT range is small, as was the case for M2CA in this study, it is reasonable for the Avg. A+B TDT values for each combination to be well-correlated with those for B-TDT.

The TDT data were also examined in various subsets. For combinations in which B was an SN2 reactive agent, the predictive equation was: Avg. A+B TDT = 54.310 + (0.492 * B-TDT); n = 13, r^2^ = 0.998, SEE = 1.044, p < 0.001. When B was not an SN2 agent the predictive equation was: Avg. A+B TDT = 53.712 + (0.499 * B-TDT); n = 17, r^2^ = 0.998, SEE = 1.242, p < 0.001. These two equations have only miniscule differences in slope, intercept and coefficient of determination compared with those of the full data set equation (see previous paragraph).

The results of the TDT regression analyses suggest that tests of binary mixtures that evaluate chemical reactivity mechanism, relative reactivity, and time-dependent toxicity of soft electrophiles, show that the simple linear relations of B-TDT with Avg. A+B TDT, provide an effective means of predicting mixture toxicity, as they account for differences in the rate of the reactive chemical, independent of the electro(nucleo)philic mechanism. The results suggest that when feasible TDT data collection should be undertaken in mixture toxicity studies.

### 3.5 Multiple linear regression-derived equations for estimating binary mixture toxicity

**Fig. 6:**
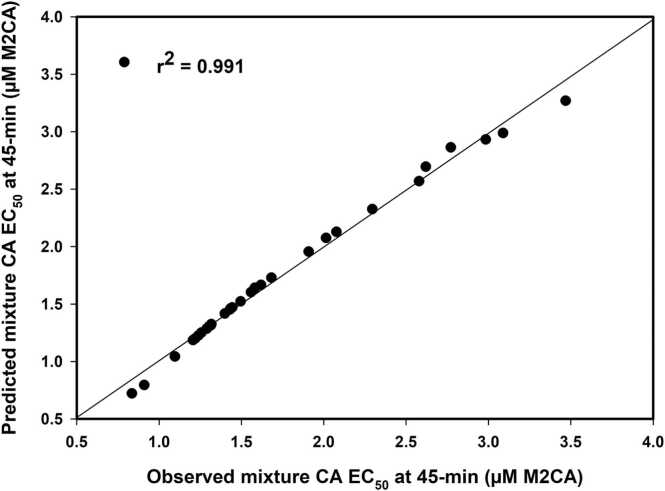
Linear regression plot of observed vs. predicted mixture toxicity for each of the 30 binary combinations. The 45-min CA EC_50_ and AQ_50_ values generated in this study were inserted into the multiple linear regression-derived equation: MX EC_50_ = -1.812 + (1.183 * CA EC_50_) + (1.572 * AQ_50_) (see Table 6) to generate the predicted 45-min MX EC_50_ value for each combination. The predicted MX EC_50_ values were then plotted against the observed 45-min MX EC_50_ values.

As in a previous report [7], MLR equations were generated for both the IA and CA models at each EC_x_ level and exposure duration (Table 6, Fig. 6). Each of the eighteen equations produced an r^2^ value > 0.960 and a VIF < 1.6, thereby being of sufficiently high quality for estimating mixture toxicity of M2CA-containing binary mixtures that were not tested herein. For any additional B of interest, one would simply need the M2CA data and the B data (converted to A-equivalent concentrations) at the appropriate exposure duration. An AQ_x_ or IQ_x_ value of interest inserted into the MLR equation then allows one to obtain an estimated MX EC_x_ value for that exposure duration.

**Table 6:** Equations for estimating binary mixture toxicity for M2CA-containing combinations.

Time^1^	IA – MLR^a^ Equations	r^2^	SEE	VIF
15	MX EC_25_ = −1.627 + (1.068 * IA EC_25_) + (1.495 * IQ_25_)	0.966	0.094	1.115
30	MX EC_25_ = −0.957 + (1.127 * IA EC_25_) + (0.847 * IQ_25_)	0.978	0.049	1.004
45	MX EC_25_ = −0.706 + (1.160 * IA EC_25_) + (0.617 * IQ_25_)	0.987	0.031	1.062
15	MX EC_50_ = −4.280 + (1.109 * IA EC_50_) + (3.825 * IQ_50_)	0.984	0.151	1.041
30	MX EC_50_ = −2.595 + (1.174 * IA EC_50_) + (2.235 * IQ_50_)	0.991	0.084	1.075
45 f	MX EC_50_ = −1.867 + (1.217 * IA EC_50_) + (1.567 * IQ_50_)	0.992	0.065	1.247
45tr	MX EC_50_ = −1.916 + (1.116 * IA EC_50_) + (1.669 * IQ_50_)	0.992	0.059	1.268
45ts	MX EC_50_ = −1.858 + (1.308 * IA EC_50_) + (1.461 * IQ_50_)	0.994	0.073	1.310
15	MX EC_75_ = −9.370 + (1.158 * IA EC_75_) + (8.101 * IQ_75)_	0.983	0.430	1.005
30	MX EC_75_ = −6.424 + (1.338 * IA EC_75_) + (4.900 * IQ_75_)	0.989	0.252	1.303
45	MX EC_75_ = −4.631 + (1.407 * IA EC_75_) + (3.399 * IQ_75_)	0.990	0.196	1.515
Time	CA – MLR Equations	r^2^	SEE	VIF
15	MX EC_25_ = −1.629 + (1.054 * CA EC_25_) + (1.537 * AQ_25_)	0.981	0.071	1.016
30	MX EC_25_ = −0.958 + (1.073 * CA EC_25_) + (0.902 * AQ_25_)	0.990	0.033	1.086
45	MX EC_25_ = −0.698 + (1.090 * CA EC_25_) + (0.653 * AQ_25_)	0.993	0.023	1.364
15	MX EC_50_ = −4.398 + (1.042 * CA EC_50_) + (4.205 * AQ_50_)	0.993	0.103	1.006
30	MX EC_50_ = −2.528 + (1.100 * CA EC_50_) + (2.336 * AQ_50_)	0.991	0.080	1.265
45 f	MX EC_50_ = −1.812 + (1.183 * CA EC_50_) + (1.572 * AQ_50_)	0.991	0.067	1.545
45tr	MX EC_50_ = −1.682 + (1.095 * CA EC_50_) + (1.731 * AQ_50_)	0.991	0.064	1.776
45ts	MX EC_50_ = −1.790 + (1.266 * CA EC_50_) + (1.454 * AQ_50_)	0.996	0.062	1.385
15	MX EC_75_ = −10.025 + (1.034 * CA EC_75_) + (9.709 * AQ_75_)	0.986	0.392	1.013
30	MX EC_75_ = −6.578 + (1.212 * CA EC_75_) + (5.552 * AQ_75_)	0.988	0.262	1.289
45	MX EC_75_ = −4.615 + (1.313 * CA EC_75_) + (3.632 * AQ_75_)	0.988	0.216	1.530

^a^ MLR – multiple linear regression; r^2^ = coefficient of determination; SEE – standard error of estimate; VIF – variance inflation factor; f – full set (n = 30); tr – training set (n = 20); ts – test set (n = 10)

Included with Table 6 are the results of validation tests of the 45-min EC_50_ MLR equations for IA and CA. Validation tests divide the data into two groups, a training set and a test set. Since there were 30 combinations, data for 20 were assigned to the training set and data for 10 to the test set. Combination selection was performed as follows. From the test chemical listing in Table 1, which is ordered by increasing level of B-TDT values, chemicals 1, 3, 4, 6, 7, 9, 10, 12 etc. were assigned to the training set and 2, 5, 8, 11 etc. to the test set. These selections were made so that each set equably spanned the spectrum of B-TDT values and chemical reactivity mechanisms. For the training and test set equations, one will note small differences in slope and intercept values for training and test set equations vs. the respective IA and CA equations for the full sets. Also, the r^2^, SEE and VIF values change only slightly across training, test and full sets. The full sets are thereby validated.

While the equations presented directly apply to *Allovibrio fischeri*, the concept is adaptable to other model organisms used in mixture toxicity testing. This assumes that cost and throughput make such efforts feasible and that high-quality CRC data with low variability are obtained. The added benefits of the approach are that the equations developed in this study span the spectrum from low-level synergism, through IA and CA, to antagonism and that various mechanisms of chemical reactivity can be evaluated with a given chemical A.

While the Microtox system has been used for these studies, this bacterial assay is used primarily for ecotoxicology assessments of potentially contaminated aquatic environments. Relevancy of these data to higher organisms is likely to be low. *De novo* use of the MLR approach will entail considerable toxicity testing. For higher organisms, it will be expensive and time-consuming. However, the results and equations generated in this study, have been presented herein to highlight the approach. This methodology has value without further testing in the following scenario:

a) From similar binary mixture studies (i.e., a common A with various B agents; likely n ≥ 10) with higher organisms from data already in-hand (e.g., in industry), one could generate MLR equations for those data.
b) Once an equation is developed, one could apply already available single chemical toxicity data for additional ‘B’ chemicals of interest.
c) For any such chemical B not included in the equation, one can take the A-alone data and the B-alone data in order to develop predicted CA and/or IA CRCs for that combination.
d) Once predicted CA and IA CRCs are computed, one can insert *any* desired AQ or IQ value into the equation to estimate mixture toxicity for that chemical A with that chemical B. For example, one might plug into the equation AQ or IQ values such as 0.75, 1.00, 1.20 and 1.50, to estimate the chemical A-equivalent mixture concentrations at which the desired toxic effect levels occur.

It is unclear whether such data are available.

### 3.6 Data availability

The raw data and additional endpoint value data, as well as SigmaPlot transforms used for analyses are available at Open Science Framework and may be accessed using the following link: https://osf.io/ysxrb.

## 4 Conclusions

This study produced the following findings. Test-to-test variability for M2CA was low. Combined effects of the mixtures included instances of toxicity that were slightly greater than predicted by IA and/or CA, consistent with CA and/or IA, coincident with CA, less toxic than predicted by one or both models, or antagonistic. In simple linear regression analyses, MX EC_50_ values were not strongly correlated with A EC_50_, B EC_50_, AQ_50_ or IQ_50_ values; however, very strong correlations were observed between B-TDT and Avg. A+B TDT data. Using MLR, predictive equations were developed from strong correlations between MX EC_x_ values and either CA EC_x_ and AQ_x_ values or IA EC_x_ and IQ_x_ values. The results demonstrate that mixture toxicity testing that incorporates simple and multiple linear regression analyses while considering chemical reactivity mechanisms, relative reactivity, and time-dependent toxicity data provides an effective means of predicting binary mixture toxicity.

## CRediT authorship contribution statement

**Schultz T. Wayne:** Writing – review & editing, Validation, Methodology, Formal analysis, Conceptualization. **Dawson Douglas Alan:** Writing – original draft, Validation, Supervision, Resources, Project administration, Methodology, Investigation, Formal analysis, Data curation, Conceptualization.

## Declaration of Competing Interest

The authors declare that they have no known competing financial interests or personal relationships that could have appeared to influence the work reported in this paper.
